# Implementation and Evaluation of Spatial Attention Mechanism in Apricot Disease Detection Using Adaptive Sampling Latent Variable Network

**DOI:** 10.3390/plants13121681

**Published:** 2024-06-18

**Authors:** Bingyuan Han, Peiyan Duan, Chengcheng Zhou, Xiaotong Su, Ziyan Yang, Shutian Zhou, Mengxue Ji, Yucen Xie, Jianjun Chen, Chunli Lv

**Affiliations:** China Agricultural University, Beijing 100083, China; hanby@cau.edu.cn (B.H.); 2021307150211@cau.edu.cn (P.D.); zhoucc@cau.edu.cn (C.Z.); 2023319010204@cau.edu.cn (Z.Y.); 2022310030218@cau.edu.cn (S.Z.); 2023308130430@cau.edu.cn (M.J.); 2023308250226@cau.edu.cn (Y.X.); chenjj@xiaotong.com.cn (J.C.)

**Keywords:** deep learning in agriculture, adaptive sampling network, precision agriculture, model lightweight deployment, apricot disease detection

## Abstract

In this study, an advanced method for apricot tree disease detection is proposed that integrates deep learning technologies with various data augmentation strategies to significantly enhance the accuracy and efficiency of disease detection. A comprehensive framework based on the adaptive sampling latent variable network (ASLVN) and the spatial state attention mechanism was developed with the aim of enhancing the model’s capability to capture characteristics of apricot tree diseases while ensuring its applicability on edge devices through model lightweighting techniques. Experimental results demonstrated significant improvements in precision, recall, accuracy, and mean average precision (mAP). Specifically, precision was 0.92, recall was 0.89, accuracy was 0.90, and mAP was 0.91, surpassing traditional models such as YOLOv5, YOLOv8, RetinaNet, EfficientDet, and DEtection TRansformer (DETR). Furthermore, through ablation studies, the critical roles of ASLVN and the spatial state attention mechanism in enhancing detection performance were validated. These experiments not only showcased the contributions of each component for improving model performance but also highlighted the method’s capability to address the challenges of apricot tree disease detection in complex environments. Eight types of apricot tree diseases were detected, including Powdery Mildew and Brown Rot, representing a technological breakthrough. The findings provide robust technical support for disease management in actual agricultural production and offer broad application prospects.

## 1. Introduction

In modern agricultural production, timely and accurate disease detection is crucial for ensuring healthy crop growth and increasing yield [1]. This is particularly vital for economically significant fruit trees such as apricot trees [2]. Traditional disease detection methods rely on the judgment of agricultural experts, which is not only inefficient but also difficult to scale across extensive farmlands [3,4]. For instance, near-infrared (NIR) spectroscopy, based on the absorption of electromagnetic radiation at near-infrared wavelengths, is widely used for the classification and detection of chemical properties in grains and nut products, facilitating quality and process control [5]. These methods are costly and inefficient and thus fail to meet practical production needs.

With the rapid development of artificial intelligence technologies, employing computer vision for disease detection has emerged as an effective solution. Garima et al. [6] introduced a convolutional neural network (CNN)-based plant disease detection method that achieved a test accuracy of 88.80%, yet the remaining 12.20% indicates room for improvement. Deepalakshmi et al. [7] utilized the LeNet architecture to create a model capable of extracting leaf features and classifying diseases under various conditions with an accuracy of 99.32%. Building on this, M. Nandhini et al. [8] proposed a gated recurrent convolutional neural network, which lacks a sufficient number of image series (G-RecConNN). Xu et al. [9] introduced a fusion of deep learning algorithms, combining residual channel attention blocks (RCABs), feedback blocks (FBs), elliptical metric learning (EML), and a CNN, termed RFE-CNN, which compensated for dataset inadequacies. Zhang et al. [10] proposed the use of pixel-weighted fusion to merge two segmentation results, enhancing the model’s robustness and segmentation performance, but they failed to utilize the 3D information effectively in the dataset. Lin et al. [11] introduced a morphologically adjusted global transformer segmentation network that improved edge recognition, with the proposed method achieving 0.903, though it still requires enhancement in segmenting small-scale objects. These existing technologies still face challenges with accuracy and generalization capabilities, especially in complex agricultural environments.

Recently, the development of attention mechanism models has offered new avenues for addressing these challenges. By integrating data from various sources, such as image data, attention mechanism techniques can provide richer and more comprehensive information, thereby enhancing the accuracy of disease detection. Elidan et al. [12] proposed latent variable networks that circumvent local maxima by combining information theoretic smoothing terms with continuation processes. Yan et al. [13] introduced a new spatial–temporal attention mechanism (STAT), which successfully considered spatial structures and automatically selected relevant time segments for predicting words, achieving optimal performance at the time but not modeling the relationships between local features, which needs further enhancement. Xie et al. [14] developed a spatial-attention-mechanism-based object tracking algorithm and established a more robust target appearance model by merging spatial attention maps with convolutional features based on the color histogram of the current frame. Sun et al. [15] proposed an encoder–decoder network that fuses spatial attention and spectral channel attention, achieving higher performance in hyperspectral image classification yet still lacking when handling fine details of hyperspectral images. Huang et al. [16] designed a generic adjustable window to mitigate boundary effects and employed spatial attention mechanisms to highlight targets in low-resolution images, significantly improving detail, but the LSDCT method lacks tracking fault determination and detection mechanisms when targets encounter prolonged complete occlusion.

The rapid advancements in deep learning, particularly the application of CNN, latent variable networks, spatial state Transformers, and large models, have opened new paths for enhancing model performance and generalization capabilities. However, effectively integrating these technologies and applying them to apricot tree disease detection remains a challenge. This study aims to address the limitations of existing apricot tree disease detection methods in terms of accuracy and efficiency by proposing an innovative detection framework that combines adaptive sampling and spatial state attention mechanisms. The innovations and contributions of this work include:

1. Model design for apricot tree disease detection based on data fusion: A multimodal large model architecture tailored for apricot tree disease detection is proposed. This architecture not only considers the visual features of image data but also integrates environmental information from sensor data, achieving effective fusion and significantly enhancing the model’s accuracy.
2. Adaptive sampling latent variable network (ASLVN): A dynamic adaptive sampling latent variable network is developed that is dynamically adjusted by two main parameters. This design allows the network to adaptively adjust according to the dynamic changes in disease progression, effectively enhancing real-time detection accuracy.
3. Spatial state attention mechanism: A spatial state attention mechanism is introduced through the design of an embedded attention aggregation module that focuses on feature areas that are highly related to disease detection. This mechanism enhances the model’s sensitivity and recognition capability for apricot tree disease features, especially when dealing with complex backgrounds and various stages of disease development.

Through these innovative methods, this study not only improves the efficiency and accuracy of apricot tree disease detection but also enhances the model’s application potential, providing robust support for disease management and food safety assurance in agricultural production.

## 2. Theoretical Foundations of the Algorithms Used

### 2.1. CNN-Based Object Detection Models

#### 2.1.1. Single-Stage Models

Object detection, a computer vision task aimed at classifying and locating objects in images or videos, has seen widespread application across various fields, including the detection of diseases in apricot trees within agricultural settings using CNNs [17,18]. Single-stage models, known for their speed and efficiency in object detection, achieve this by predicting object locations and categories in a single forward pass.

The You Only Look Once (YOLO) model [19] divides the input image into an S×S grid, where each cell predicts multiple bounding boxes and their corresponding class probabilities to effectively detect objects. Each grid cell predicts *B* bounding boxes [20], with each defined by five parameters: center coordinates (x,y), width *w*, height *h*, and the confidence score *c* of the bounding box containing an object. Additionally, each cell predicts a probability distribution over *C* classes, resulting in B×5+C parameters per grid cell describing potential objects and their categories. The YOLO model’s loss function consists of three components: coordinate loss, confidence loss, and classification loss. The total loss function for the YOLO model is given by:

(1) $$L_{\mathrm{total}}=L_{\mathrm{coord}}+L_{\mathrm{conf}}+L_{\mathrm{class}}$$

This design enables YOLO to detect all objects in an image within a single forward pass, achieving rapid detection [21]. The Single-Shot MultiBox Detector (SSD) model adopts a similar approach but differs in its design by utilizing convolutional feature maps of various scales to predict bounding boxes of different sizes, thus addressing multi-scale object detection challenges [22]. SSD predicts a fixed number of bounding boxes per feature map, each comprising location coordinates, dimensions, confidence, and class probabilities. Like YOLO, SSD’s loss function includes location and confidence losses. Location loss is calculated as follows:

(2) $$L_{\mathrm{loc}}=\sum_{i}m_{i}\sum_{j\in \{x,y,w,h\}}\mathrm{SmoothL}1\left(p_{i}^{j}-g_{i}^{j}\right)$$

where SmoothL1 is a smoothing function that reduces the impact of outliers, $m_{i}$ indicates whether the *i*-th predicted box contains an object, and $p_{i}^{j}$ and $g_{i}^{j}$ represent the predicted and actual parameters, respectively. Confidence loss evaluates the discrepancy between the predicted class probabilities and the actual classes:

(3) $$L_{\mathrm{conf}}=-\sum_{i}m_{i}\log\left(p_{i}^{c_{i}}\right)-\sum_{i}\left(1-m_{i}\right)\log\left(p_{i}^{0}\right)$$

where $p_{i}^{c_{i}}$ and $p_{i}^{0}$ represent the probabilities of the actual class and no object being present, respectively. Combining these losses, SSD achieves fast and accurate object detection, effectively handling multi-scale challenges.

In summary, both YOLO and SSD, through their unique designs, facilitate rapid object detection and are particularly valuable in the context of apricot tree disease detection. YOLO’s single forward pass makes it suitable for real-time applications, whereas SSD’s ability to handle multiple scales allows for effective detection of diseases on different parts of an apricot tree. Both models incorporate well-designed loss functions that enhance detection accuracy and overall performance. By integrating these single-stage models, improved results in apricot tree disease detection can be achieved [23,24,25].

#### 2.1.2. Two-Stage Models

In computer vision tasks, two-stage object detection models achieve target localization and classification through two distinct phases [26]. The Faster R-CNN model, for instance, plays a crucial role in object detection across various fields by providing an efficient and accurate solution [27]. In the agricultural domain, this model is employed for detecting diseases in apricot trees, thereby aiding farmers and agricultural professionals with timely disease identification and management [28].

Faster R-CNN, a typical two-stage object detection model, features a region proposal network (RPN) as a key component of its first stage [29]. Initially, Faster R-CNN extracts features from the input image through convolutional layers, creating a feature map. Subsequently, the RPN generates a series of region proposals on this feature map. The loss function of the RPN comprises two components: classification loss and localization loss:

(4) $$L_{\mathrm{RPN}}=\frac{1}{N_{\mathrm{cls}}}\sum_{i}L_{\mathrm{cls}}\left(p_{i},p_{i}^{*}\right)+\frac{1}{N_{\mathrm{reg}}}\sum_{i}p_{i}^{*}L_{\mathrm{reg}}\left(t_{i},t_{i}^{*}\right)$$

where $L_{\mathrm{cls}}$ measures whether a target is present within the proposed box, and $L_{\mathrm{reg}}$ quantifies the discrepancy between the proposed and actual bounding box dimensions. Here, $p_{i}$ and $p_{i}^{*}$ denote the predicted and actual probabilities of an object’s presence, respectively, while $t_{i}$ and $t_{i}^{*}$ represent the predicted and actual bounding box parameters. $N_{\mathrm{cls}}$ and $N_{\mathrm{reg}}$ serve as normalization factors for the classification and localization losses, respectively.

Following the generation of region proposals by the RPN, the second stage fine-tunes these proposals by performing precise classification and regression [30]. The feature map along with the region proposals are forwarded to a fully connected layer, where each proposal is classified and its position is adjusted to produce the final detection outcomes. The loss function for the second stage also includes classification and localization losses; the overall loss function is a weighted sum of the losses from both stages:

(5) $$L_{\mathrm{total}}=L_{\mathrm{RPN}}+L_{\det}$$

Through its two-stage design, Faster R-CNN facilitates efficient and precise object detection [31]. In the context of apricot tree disease detection, the model uses the RPN to generate initial proposals, which are then classified in the second stage to identify diseases. The high accuracy of this model helps with the early detection of diseases, preventing their spread. Depending on the specific requirements of apricot tree disease detection, adjustments can be made to the Faster R-CNN model.

### 2.2. Attention Mechanisms

In the field of deep learning, attention mechanisms have emerged as a critical technology that enhances the model’s capability to process diverse inputs. These mechanisms enable more precise feature detection in models, thereby significantly improving the accuracy of disease detection in apricot trees [32,33]. The fundamental concept behind attention mechanisms is the computation of the importance of different parts within the input and the assigning of variable weights to these parts, which thus allows the model to focus more effectively on critical sections [34]. One common type of attention mechanism is the self-attention mechanism [35], which calculates the relationships between each element in the input sequence and every other element and assigns varying weights to enhance the model’s understanding of the input. The self-attention mechanism can be expressed by the following mathematical formula:

(6) $$\mathrm{Attention}\left(Q,K,V\right)=\mathrm{softmax}\left(\frac{QK^{T}}{\sqrt{d_{k}}}\right)V$$

where *Q*, *K*, and *V* represent the query, key, and value vectors, respectively, and $d_{k}$ is the dimensionality of the key vectors. This formula allows the model to calculate the degree of association between elements within the input, assigning appropriate weights accordingly.

Another prevalent form of attention is channel attention [36], which allocates different weights to various channels of an image during the feature extraction phase, thereby enabling the model to prioritize significant channels. The channel-attention mechanism typically incorporates operations such as global average pooling (GAP) and global max pooling (GMP) to aggregate features across different channels. The core formula for channel attention is given by:

(7) $$\alpha_{c}=\sigma \left(W_{1}\left(\mathrm{GAP}\left(X\right)\right)+W_{2}\left(\mathrm{GMP}\left(X\right)\right)\right)$$

where $\alpha_{c}$ denotes the attention weight for channel *c*, σ represents an activation function, such as sigmoid, and $W_{1}$ and $W_{2}$ are fully connected layers that process the outputs from the GAP and GMP operations, respectively.

By incorporating these attention mechanisms, apricot tree disease detection models are better able to extract crucial features from images [37]. For instance, self attention allows the model to focus on areas within the image where diseases are present, while channel attention focuses on features related to disease, such as color and texture, that are specific to certain channels.

Moreover, hybrid attention mechanisms [38] can be employed to enhance the model’s capability to handle multimodal data by combining self attention and channel attention. This involves using image and sensor data as inputs and employing self attention to compute correlations between data points, followed by channel attention to assign weights to different channels, thereby improving the model’s understanding of multimodal data.

The loss function for attention mechanisms can be tailored to specific applications. In apricot tree disease detection, the model’s performance can be evaluated using the following loss function:

(8) $$L_{\mathrm{total}}=L_{\det}+\lambda_{\mathrm{att}}L_{\mathrm{att}}$$

where $L_{\det}$ is the detection loss that measures the model’s capability in classifying and localizing targets, $L_{\mathrm{att}}$ is the attention loss that evaluates the contribution of the attention mechanism to model performance, and $\lambda_{\mathrm{att}}$ is a weighting factor that adjusts the influence of attention loss in the overall loss. This careful design ensures that while the attention mechanism enhances model performance, it does not unduly complicate the model, thus maintaining accuracy and efficiency in detecting apricot tree diseases.

### 2.3. Deep Learning Model Lightweighting

As the application of deep learning technology has expanded across various domains, the complexity of models and their demand for computational resources have correspondingly increased. In the field of agriculture, such as for apricot tree disease detection, model lightweighting is particularly crucial due to the often resource-constrained environments, where real-time or near-real-time detection is required [39,40]. Lightweighting a design maintains performance while reducing resource consumption and deployment costs, thus achieving more efficient disease detection. Lightweighting of deep learning models can be accomplished through various methods, including model pruning, quantization, structural optimization, and knowledge distillation.

Model pruning effectively reduces the quantity of parameters within a model by removing unnecessary or redundant neurons and connections, thus simplifying the model structure [41]. Pruning can be categorized into static and dynamic types. Static pruning is performed post-training and removes connections with minimal weights or minor contributions to model performance; dynamic pruning adjusts neurons and connections in real-time based on the model’s operational state during training. Pruning effectively reduces the model’s parameter count and computational load, making it more suitable for resource-limited settings.

Quantization is another common lightweighting method and involves converting model parameters and computations from floating-point to fixed-point numbers, thereby reducing the model’s memory usage and computational demands [42]. Quantization can be implemented post-training or concurrently with training (quantization-aware training). In the context of apricot tree disease detection, quantization-aware training enables the model to retain performance while reducing computational load, allowing operation on low-power devices. Quantization significantly lowers the computational load during the model’s inference phase, facilitating operation on low-power devices.

Structural optimization is an essential aspect of lightweight design, where lightweight model structures, such as MobileNet and EfficientNet, are introduced [43,44,45]. MobileNet utilizes depthwise separable convolutions to reduce the computational load of convolutional layers while maintaining performance [46]. This convolution type comprises depthwise convolutions, which apply a single filter per input channel, and pointwise convolutions, which use a 1×1 convolution to combine the features across channels, thus substantially reducing the computational demand.

Knowledge distillation is a method whereby complex model knowledge is transferred to a simplified model [47]. By having the lightweight model learn the output distribution of a more complex model, it is possible to maintain performance while reducing complexity. The loss function for knowledge distillation includes basic loss and distillation loss:

(9) $$L_{\mathrm{distill}}=L_{\mathrm{base}}+\lambda_{\mathrm{distill}}\sum_{i}KL\left(p_{i},q_{i}\right)$$

where KL is the Kullback–Leibler divergence, which measures the discrepancy between the output distributions of the lightweight model $q_{i}$ and the complex model $p_{i}$, and $\lambda_{\mathrm{distill}}$ is a weighting factor for the distillation loss. Through knowledge distillation, the model complexity is effectively reduced, enhancing its practicality for apricot tree disease detection.

By employing these lightweighting techniques, models suitable for apricot tree disease detection can be designed to perform accurately and efficiently in resource-limited environments [48]. Future research may further explore how to combine these methods to achieve even better lightweighting effects.

## 3. Materials and Method

### 3.1. Dataset Collection

**Figure 1:**
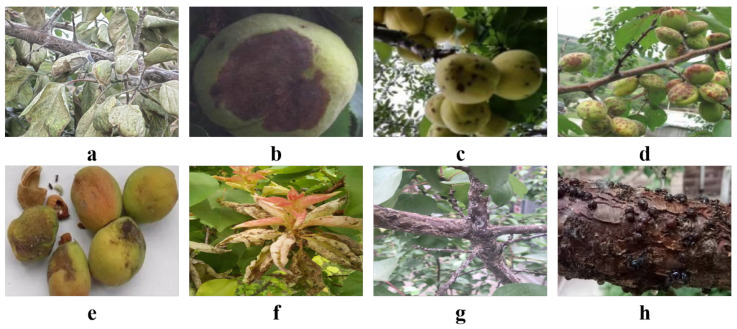
Samples from the dataset used in this paper: (**a**) Powdery Mildew Disease, (**b**) Brown Rot Disease, (**c**) Scab Disease, (**d**) Bacterial Leaf Spot Disease, (**e**) Almond Bee Disease, (**f**) Apricot Sore Disease, (**g**) Scale Chosomiasis Disease, and (**h**) Apricot Moth Disease.

The dataset utilized in this study was collected from rural areas in Bayannur’s Linhe District, Urad Front Banner in the Inner Mongolia Autonomous Region, Changping District in Beijing, and Yongqing County in Langfang, Hebei Province, and was supplemented by data crawled from the internet. Several villages cultivating apricot trees were selected for months-long field research and data collection activities. Throughout the data collection process, professional digital cameras and smartphone cameras were employed to capture images. The camera models included Canon EOS series and Nikon D series, ensuring high-resolution imaging, while smartphone models from the Samsung Galaxy and Apple iPhone series facilitated a broader range of preliminary detection and collection. Data collection was conducted during the spring, summer, and autumn seasons of 2023. Special attention was paid to each season to better capture the manifestation of apricot tree diseases across different growth phases, particularly during the summer and autumn when disease symptoms were most prominent. Several representative apricot orchards were chosen for intensive study, and each apricot tree within these areas was thoroughly examined and documented, including details such as tree age, environmental conditions, and type and severity of diseases, thereby creating a comprehensive profile for each tree. For each disease type, a large volume of images was collected that captured multiple instances of the disease from various angles, distances, and lighting conditions to ensure data diversity and comprehensiveness, as shown in Figure 1.

Brown Rot Disease primarily affects apricot fruits and flowers and is characterized by the appearance of water-soaked, brownish, soft rot spots on the fruit surface, leading to rapid decay of the entire fruit under severe conditions. This disease progresses swiftly in humid conditions and is easily transmitted during storage. Powdery Mildew Disease is mainly manifested by the presence of a white powdery substance on apricot leaves, tender branches, and fruits. This disease causes leaf deformation, desiccation, and eventual shedding, severely impacting the tree’s photosynthesis and fruit quality. Scab Disease causes round to irregular dark brown spots on leaves and fruits, leading to premature leaf drop under severe conditions and damaging the appearance and quality of the fruit and affecting market value. Bacterial Leaf Spot Disease is caused by bacteria and leads to small perforations in the leaves, with the centers of the spots gradually falling out to form holes, severely affecting the growth and development of apricot trees. Almond Bee Disease is caused by a type of wasp and primarily harms the fruit, causing internal damage and affecting the internal quality and storage capability of the fruit. Apricot Sore Disease is characterized by the appearance of ulcerative cankers on the bark, followed by cracking or even shedding of the bark, severely affecting the tree’s vascular system and overall health. Scale Chosomiasis Disease sucks sap from apricot trees, causing leaf yellowing and slow growth, which can lead to branch desiccation in severe cases. These pests also secrete honeydew, which induces sooty mold, further damaging the trees. Apricot Moth Disease mainly harms the leaves and tender shoots of apricot trees, sucking plant sap, which leads to deformed leaves and restricted growth, severely impacting the overall growth of the trees. Detailed data on different disease types, including the number of samples and locations, are outlined in Table 1.

Additionally, in this study, sensor data were integrated as auxiliary information into the disease detection model. The types of sensors used and their specific applications are as follows. We mainly used the following types of sensors:

1. Environmental sensors: These sensors monitor environmental parameters such as temperature, humidity, and light intensity. These data help analyze the conditions under which diseases occur and develop.
2. Spectral sensors: These sensors capture the spectral information of plant leaves. By analyzing the reflectance of leaves at different wavelengths, it is possible to detect internal disease conditions within the leaves.
3. Near-infrared sensors: These sensors acquire near-infrared images of plants, which are particularly effective for early disease detection because near-infrared light can penetrate the leaf surface and detect potentially diseased areas.

In the field experiments, these sensors were installed in different plots to monitor the environment and the state of the plants in real-time. All sensor data were transmitted via wireless network to a central database for unified management and analysis. During model training, sensor data were integrated with image data to form multimodal data inputs. Specifically, environmental data and spectral data were used as additional features that were input into the deep learning model and worked together with image features to improve detection accuracy. For spectral data, feature extraction algorithms were employed to convert the spectral reflectance at different wavelengths into feature vectors that could be integrated with the image data.

### 3.2. Dataset Annotation

In the study of apricot disease detection, accurately determining and annotating disease stages is crucial for the training and evaluation of the model. We divided apricot diseases into several main stages, including early, middle, and late stages. Regarding the disease stage classification standards, we based the divisions on the pathological characteristics and development process of the diseases. The early stage typically manifests as a few small spots or slight discoloration on the leaves; the middle stage shows enlarged and gradually merging spots, with noticeable leaf lesions; the late stage is characterized by large areas of spots, severe leaf damage, and even wilting and falling off. For specific annotations and validation, we invited several plant pathology experts to participate in the data annotation work. These experts conducted detailed analyses of the visual characteristics, spot morphology, and development stages of apricot leaves to determine the specific disease stage in each image. To ensure the accuracy and consistency of the annotations, all results underwent multiple reviews and cross-validation. Additionally, we used the LabelMe image annotation tool (http://labelme2.csail.mit.edu/Release3.0/index.php), which allowed annotators to mark disease spots on the images and select the corresponding disease stage labels. All annotation data were systematically stored in a database, facilitating subsequent data processing and model training.

### 3.3. Dataset Enhancement

**Figure 2:**
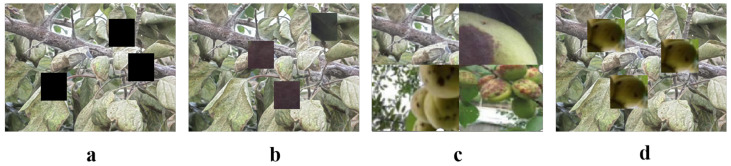
Image dataset enhancement methods used in this paper: (**a**) Cutout, (**b**) Cutmix (During training, the labels of the target objects can be weighted according to the mixing ratio of the regions. For example, if the proportions of the original image and the mixed image are λ and 1−λ, respectively, the label weights of the target objects can be adjusted accordingly to λ and 1−λ), (**c**) Mosaic, (**d**) Replication Augmentation (The duplicated target objects should retain the same label information as the original objects. In the annotation file, all duplicated target objects should have labels consistent with the original objects to ensure that the model can correctly identify and classify these target objects during training).

In the task of detecting diseases in apricot trees, the adoption of data augmentation techniques such as Cutout, Cutmix, Mosaic, and copy enhancement is crucial, as shown in Figure 2. These methods significantly enhance the diversity and complexity of the model training data by simulating various visual changes and occlusion scenarios, aiding with improving the model’s ability to recognize apricot diseases in real-world environments. Particularly in cases of limited data, these techniques can effectively expand the dataset to enhance the model’s generalization ability, reduce overfitting, and thereby increase the accuracy and robustness of the model in practical applications.

#### 3.3.1. Cutout

Cutout is regarded as an effective image data augmentation technique and is particularly suitable for training deep learning models to enhance their ability to recognize obstructed objects [49]. In applications such as apricot tree disease detection, the Cutout technique helps models accurately identify diseases even when leaves are partially visible or obstructed. During implementation, a rectangular region in the image is randomly selected at the training stage, and the pixel values within this region are set to zero or replaced with the average pixel value of the image. This treatment not only simulates occlusion but also enhances the model’s reliance on other information within the image, thus improving the model’s adaptability to complex backgrounds or incomplete information. Mathematically, the Cutout operation can be represented as:

(10) $$I^{\prime }=I⊙\left(1-M\right)+m\cdot M$$

Here, *I* is the original image, $I^{\prime }$ is the image processed by Cutout, and ⊙ represents element-wise multiplication. *M* is a binary mask of the same size as the image *I*, where elements within the selected rectangular area are set to 1, and the rest of the image is set to 0; *m* is a scalar representing the pixel value used to fill the obstructed area and is typically chosen as 0 or the global average pixel value of the image *I*.

In the context of apricot tree disease detection, the Cutout technique simulates situations where leaves might be partially covered by other leaves or obstacles. This challenges the model to identify the presence and type of disease based on partial information. Consequently, Cutout not only enhances the model’s generalization ability but also improves its practicality in agricultural environments by preventing overfitting and enhancing the model’s adaptability.

#### 3.3.2. Cutmix

Cutmix is a widely used image data augmentation technique that combines parts of two images to generate a new image [50], increasing data diversity and enhancing the model’s generalization ability. This method is particularly suitable for complex image recognition tasks such as apricot tree disease detection as it not only enhances the model’s ability to recognize disease features against various backgrounds but also aids with accurate judgment and classification in different environments. In practice, Cutmix randomly selects a rectangular area from one image and pastes it onto the same position in another image. This operation involves the mixing of pixels and labels by adjusting the proportion of label contributions. If the area cut from the first image represents a proportion λ of the entire image, the label of the newly generated image is also mixed in this proportion. Mathematically, the Cutmix operation can be expressed as:

(11) $${\mathrm{Image}}_{\mathrm{new}}=M⊙{\mathrm{Image}}_{1}+\left(1-M\right)⊙{\mathrm{Image}}_{2}$$

Here, ${\mathrm{Image}}_{1}$ and ${\mathrm{Image}}_{2}$ are the images selected for mixing, *M* is a binary mask for which the selected area is set to 1 and the rest of the image is set to 0, and ⊙ denotes element-wise multiplication. For label mixing, assuming the labels of the two images are ${\mathrm{Label}}_{1}$ and ${\mathrm{Label}}_{2}$, the mixed label is:

(12) $${\mathrm{Label}}_{\mathrm{new}}=\lambda \cdot {\mathrm{Label}}_{1}+\left(1-\lambda \right)\cdot {\mathrm{Label}}_{2}$$

Here, λ is typically calculated based on the area proportion of the cut region, ensuring that the proportion of label mixing corresponds with the content blending of the images. In the application of apricot tree disease detection, Cutmix provides an effective method to simulate various disease conditions that might appear on apricot leaves, such as a single leaf exhibiting multiple types of disease spots. By generating images through this method, the model encounters more complex and variable disease scenarios during training, enhancing its ability to recognize and categorize diseases in practical applications. Additionally, this technique significantly increases the size and diversity of the training dataset, helping to prevent model overfitting and boosting the model’s robustness in different environments.

#### 3.3.3. Mosaic

Mosaic data augmentation [51] is a novel image processing technique that combines regions from four different images into a single new image. In apricot tree disease detection, Mosaic enhances the model’s ability to learn disease features and improves recognition performance under various backgrounds and environmental conditions. Operationally, Mosaic data augmentation begins by randomly selecting four images from the training dataset. Then, a quarter-sized region from each image’s four different corners is cropped. These four regions are recombined to form a new, complete image. Mathematically, the Mosaic operation can be described as follows:

(13) $$I_{\mathrm{new}}=\left[\begin{array}{cc} I_{A,\mathrm{top}‐\mathrm{left}} & I_{B,\mathrm{top}‐\mathrm{right}}\\ I_{C,\mathrm{bottom}‐\mathrm{left}} & I_{D,\mathrm{bottom}‐\mathrm{right}} \end{array}\right]$$

Here, $I_{A},I_{B},I_{C},I_{D}$ are the four randomly selected images: each contributing a part to the new image $I_{\mathrm{new}}$. For example, $I_{A,\mathrm{top}‐\mathrm{left}}$ represents the top-left corner of image $I_{A}$.

In apricot tree disease detection, the Mosaic technique combines parts of images with different backgrounds and disease stages to generate new training samples, enhancing the model’s adaptability to complex backgrounds. Additionally, the new images integrate various disease features, improving the model’s multitasking capability and enabling it to handle multiple disease diagnosis tasks simultaneously in practical applications. Mosaic technology enriches the training data content, forcing the model to learn from a broader range of data features and, thereby, improving its generalization ability.

#### 3.3.4. Replication Augmentation

Replication augmentation [52] is an effective image data augmentation method that is particularly suitable for increasing the frequencies of specific features in images during the training of deep learning models, thereby helping the model better learn and recognize these features. In apricot tree disease detection tasks, this method is critical because some disease features may be rare or occupy only a small part of the image under natural conditions. The basic idea of Replication Augmentation is to select a key area in the image, such as an apricot tree leaf affected by disease, and then repeat this area in other positions within the same image. Initially, the key feature area in the image is identified, usually by relying on advanced image processing technologies such as object detection algorithms to automatically recognize and locate disease areas in the image. Once the key area is identified, it is replicated one or more times. The replication process may involve transformations such as rotation and scaling to increase sample diversity. The replicated areas are pasted into other positions of the original image. Mathematically, the operation of Replication Augmentation can be expressed by the following formula:

(14) $$I^{\prime }=I+\sum_{i=1}^{N}T_{i}\left(M\cdot I\right)$$

Here, *I* is the original image, *M* is a binary mask indicating the selected area, $T_{i}$ are the affine transformations applied to the *i*-th replicated area, including translation, rotation, and scaling, *N* is the number of times replication occurs, and $I^{\prime }$ is the augmented image.

In apricot tree disease detection, Replication Augmentation significantly improves the model’s ability to recognize rare disease features. For example, if a specific disease appears in only a few images within the dataset, Replication Augmentation can increase the presence of this disease across multiple images, providing the model with more opportunities to learn how to recognize it. Additionally, this method helps the model better understand the relationship between disease features and other background elements, allowing for accurate disease recognition even against complex or disruptive backgrounds.

### 3.4. Proposed Method

#### 3.4.1. Overview

**Figure 3:**
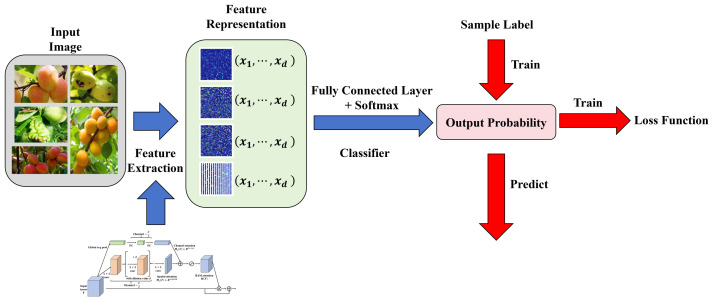
The illustration describes the architecture flow of the apricot disease detection model proposed in this paper. This process includes image input, feature extraction, classification, and the training and prediction processes. Initially, the input images are processed through a CNN for feature extraction, where the CNN layers capture the key features within the images; subsequently, the extracted feature vectors are passed to fully connected layers, which output the final probabilities of disease categories through the softmax function. During the training phase, the model optimizes parameters via the loss function, whereas in the prediction phase, the model uses these trained parameters to assess new images for disease detection. This flowchart clearly demonstrates the complete steps from image processing to disease diagnosis, effectively supporting the experimental part and theoretical analysis of the method described in this paper.

In this study, a comprehensive deep learning framework is proposed with the aim of efficiently detecting and classifying diseases in apricot trees. The design of the entire system considers the full process from model input to final decision output, including model design, the ASLVN, the spatial state attention mechanism, and the lightweight deployment of the model. Figure 3 provides a detailed description of the construction process of this method.

Initially, the pre-processed images and sensor data are input into a deep CNN, which is responsible for extracting fundamental features from the images. The model design employs a multi-level feature fusion strategy to enhance the capture of disease features in apricot trees. Specifically, the model structure incorporates residual connections and depthwise separable convolution layers, which not only accelerate the training process but also enhance the network’s perception of fine details, which is crucial for disease detection. On this basis, an ASLVN is introduced, which is a dynamic system based on hidden Markov models (HMMs) for capturing state transitions during the disease process. The ASLVN dynamically adjusts its parameters based on the features extracted from the base CNN to adapt to different stages of disease progression. Each state is not only associated with a potential disease stage but also dynamically adjusts the data sampling frequency and network parameters to optimize the learning process.

Regarding the operation of GCNs on feature maps and their integration with the ASLVN, one of the core innovations of this paper is treating each pixel in the image as a node in a graph. In this way, the GCN can be utilized to capture spatial relationships between pixels. In practical application, each pixel not only contains its color information but also carries positional information, allowing the construction of a pixel-level graph where nodes represent pixels and edges are based on spatial adjacency relationships between pixels. For instance, each pixel can be connected to its eight neighbors (left, right, up, down, and the four diagonal directions). On this basis, a GCN updates the node states by applying graph convolution operations on this graph to capture and utilize local spatial relationships. During the convolution process in a GCN, the new state of a node is derived from the weighted states of its neighbors, with weights typically determined by the connections between nodes and their respective features. This way, each node reflects not only its own characteristics but also integrates information from surrounding neighbors, which is particularly effective for understanding and analyzing spatial patterns in images.

Following feature extraction, a spatial state attention mechanism is introduced. This mechanism constructs a multi-level attention model capable of identifying and emphasizing features in key areas of the image. The attention model employs a GCN to compute dependencies between pixels in the image, achieving spatial weighting of features and enhancing model sensitivity to diseased areas. Mathematically, spatial attention can be represented as:

(15) Attention(X)=σ(GCN(X)·W)

where *X* denotes the input features, *W* is the learned weight matrix, and σ is the activation function, such as softmax, used to generate attention weights for each pixel point. In this paper, the term “state” refers to the representation state of graph nodes (i.e., pixels) at a specific processing stage. These states are influenced by the input image features and their processing history within the network. In the spatial state attention mechanism, different attention weights are assigned to nodes based on their states to highlight those regions more crucial to the current task. Specifically, the attention mechanism learns how to assign importance weights to different nodes (or regions), allowing the network to focus more on features relevant to disease detection, such as the characteristics of infected areas.

To enable the model to operate effectively on edge devices, a model lightweighting process is undertaken. Initially, redundant network parameters that contribute minimally to model performance but occupy significant computational resources are removed through network pruning techniques. Subsequently, knowledge distillation technology is applied to transfer knowledge from a complex model to a lightweight network structure. This step involves transferring knowledge from the teacher network (original large model) to the student network (lightweight model), with the loss function including:

(16) $$L=L_{\mathrm{task}}\left(y,\hat{y}\right)+\alpha \cdot KL\left(\mathrm{Softmax}\left(z/T\right),\mathrm{Softmax}\left(\hat{z}/T\right)\right)$$

Here, $L_{\mathrm{task}}$ is the task-specific loss, such as cross-entropy loss, KL is the Kullback–Leibler divergence used to measure the distribution differences between the outputs of the teacher and student networks, *T* is the temperature parameter, and α is a weight factor that adjusts the importance of the two loss components. Through this series of designs and optimizations, the proposed method not only enables real-time detection of apricot tree diseases on devices with limited computing resources but also ensures high accuracy and efficiency of detection. The entire process’s design considers every detail from data input to processing output, ensuring the system’s high performance and practicality.

#### 3.4.2. Adaptive Sampling Latent Variable Network

**Figure 4:**
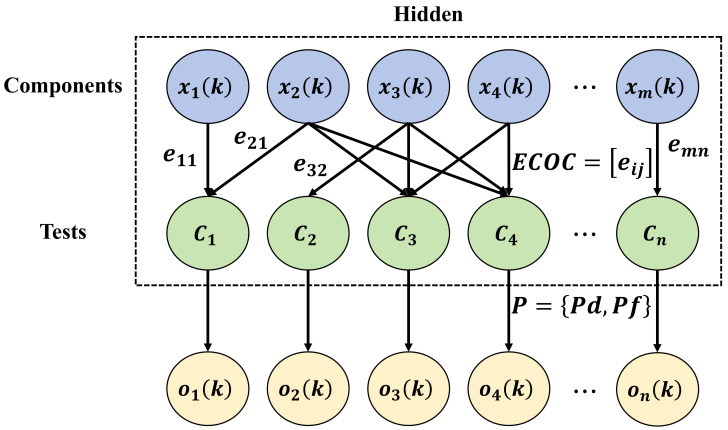
The illustration shows the conceptual model of the ASLVN (adaptive sampling latent variable network) structure proposed in this paper. It details the entire process from the input layer to the output layer and all its components, including the input data $x_{1}^{k},x_{2}^{k},\ldots ,x_{m}^{k}$ and the resulting outputs $o_{1}^{k},o_{2}^{k},\ldots ,o_{n}^{k}$ processed through the network. The diagram also shows the connections and information flow between different layers, particularly highlighting the latent variables in the hidden layers and how they impact the output results. The ASLVN structure effectively integrates data features and optimizes the learning process of the network through an adaptive sampling mechanism, enhancing the model’s ability to handle complex data structures. This structure diagram clearly expresses the core mechanism and working principles of the ASLVN, providing an intuitive view for understanding and analyzing the network structure.

In this study, an innovative ASLVN is proposed, which innovates on the traditional HMM to meet the complex requirements of apricot tree disease detection. Unlike traditional periodic sampling methods, adaptive sampling automatically adjusts the sampling frequency based on the dynamic changes in disease progression, thereby more effectively utilizing computational resources and improving the timeliness and accuracy of disease prediction, as shown in Figure 4.

Traditional sampling methods typically collect data at fixed time intervals or fixed spatial distributions, such as every hour or in each fixed area. These methods may not have timely responses to changes in dynamically changing environments, leading to information loss or processing delays. The adaptive sampling method adjusts the sampling frequency and areas dynamically by analyzing changes in the data in real-time. In this study, the implementation of adaptive sampling relies on the estimation of hidden states, which are inferred through the outputs of the latent variable network. The transition probabilities of hidden states can be expressed as:

(17) $$P\left(s_{t+1}|s_{t}\right)=\mathrm{softmax}\left(W\cdot h\left(s_{t}\right)+b\right)$$

where $s_{t}$ and $s_{t+1}$ represent the hidden states at times *t* and t+1, respectively, *W* and *b* are network parameters, and $h(s_{t})$ is the network output feature under the hidden state $s_{t}$. The core of adaptive sampling is to dynamically adjust the sampling strategy based on the state transition probabilities. Specifically, the sampling interval *T* is defined as:

(18) $$T=\frac{1}{\lambda \cdot P(s_{t+1}|s_{t})}$$

where λ is a tuning parameter used to control the sensitivity of the sampling frequency. In this way, when the model predicts a significant change in future states (i.e., $P(s_{t+1}|s_{t})$ is high), the sampling frequency will increase accordingly to capture critical change information. The design of the ASLVN employs a multi-layer neural network structure to ensure sufficient model capacity to capture complex state transitions. Specific network parameters include: The input layer: accepts feature vectors from image preprocessing with a dimension of 256. Hidden layers: consist of three fully connected layers with neuron counts of 128, 64, and 32, respectively; these layers use the ReLU activation function to increase non-linear expression capability. Output layer: outputs the transition probabilities from the current state to the next state, with dimensions matching the number of states, using the softmax function for probability normalization.

Applied to this study’s task, the ASLVN can dynamically adjust the sampling strategy to monitor the development stages of apricot tree diseases in real-time, which is particularly critical for early disease detection and treatment. Additionally, by precisely controlling the sampling frequency, this network significantly reduces the requirements for data processing and storage while ensuring detection accuracy, which is particularly important for deployment on edge devices.

#### 3.4.3. Spatial State Attention Mechanism

**Figure 5:**
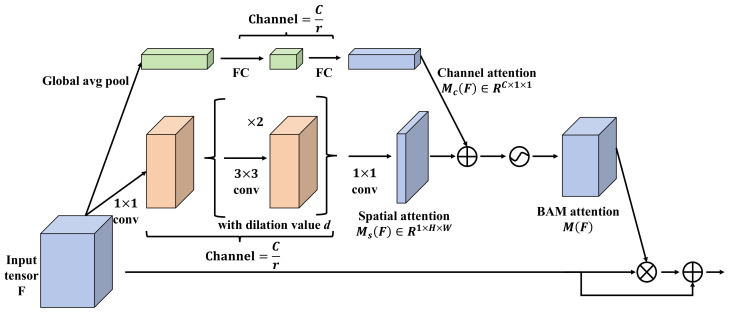
The illustration shows the architectural details of the spatial state attention mechanism proposed in this paper. This includes both channel attention and spatial attention modules, showing how the integration of these two attention mechanisms enhances the model’s focus on features. The channel attention module learns the importance of different channels through global average pooling and fully connected layers, thereby weighting the features of each channel; the spatial attention module focuses on the importance of spatial locations, emphasizing key spatial areas through convolution operations. The collaborative function of these two modules forms the spatial state attention mechanism described in this paper, effectively enhancing the model’s ability to recognize key features in apricot disease detection.

In visual tasks of deep learning, especially when dealing with complex scenes such as apricot tree disease detection, accurately focusing on key areas within an image becomes critical for enhancing model performance. Therefore, an innovative spatial state attention mechanism is introduced in this study; it is implemented through a GCN and effectively identifies and emphasizes key features within images: particularly, disease features against complex backgrounds, as shown in Figure 5.

The design of the spatial state attention mechanism employs a multi-layer graph convolutional network, which uses spatial relationships between nodes (pixels) to compute attention weights. The fundamental idea of a graph convolutional network is to perform convolution operations on graph-structured data, enabling the network to capture local connection patterns of nodes and utilize the relationships between nodes to enhance the capability of feature representation. Specifically, the spatial state attention mechanism consists of three layers of graph convolutions, with each designed to handle spatial relations at different scales in order to capture image features from local to global. The first layer of graph convolution addresses smaller neighborhoods, capturing fine-grained features; the second layer expands the receptive field, integrating mid-scale spatial information; the third layer extends further to capture broader contextual information. The parameters for each layer of graph convolution are as follows:

The first layer has input channels with a dimension of 256 and output channels with a dimension of 128, and it uses a 3 × 3 convolution kernel. The second layer has input channels with a dimension of 128 and output channels with a dimension of 64, and it uses a 5 × 5 convolution kernel. The third layer has input channels with a dimension of 64 and output channels with a dimension of 32, and it uses a 7 × 7 convolution kernel. The output of each layer undergoes a linear transformation through a weight matrix *W*, and then, the final attention weights are computed using the softmax function. The mathematical expression is:

(19) $$\mathrm{Attention}\left(X\right)=\mathrm{softmax}({\mathrm{GCN}}_{3}\left({\mathrm{GCN}}_{2}\left({\mathrm{GCN}}_{1}\left(X\right)\right)\right)\cdot W)$$

where *X* represents the input features, and ${\mathrm{GCN}}_{1},{\mathrm{GCN}}_{2},{\mathrm{GCN}}_{3}$ are the three layers of the graph convolutional network. Compared to the self-attention mechanism used in traditional Transformers, the main difference in the spatial state attention mechanism is its ability to consider spatial relationships within the image. Traditional self-attention mechanisms compute relationships between all elements in a fully connected manner, whereas the spatial state attention mechanism focuses on locally connected pixels through graph convolutions. This approach is more natural and effective when handling spatial data such as images, particularly for images where the target structure is complex and closely related to the environmental background. The main advantage of employing a spatial state attention mechanism lies in its excellent adaptability to environments and its ability to enhance key features. In the task of apricot tree disease detection, diseases often affect only a small portion of leaves or fruits, and these areas may visually resemble healthy areas. The spatial state attention mechanism effectively highlights the features of these key areas, thereby aiding the model with more accurately locating and identifying diseases. Moreover, by capturing spatial information at different levels, this mechanism also enhances the model’s robustness to complex backgrounds, maintaining high recognition accuracy in various field environments.

#### 3.4.4. Model Lightweight Deployment

In the implementation of deep learning models for detecting diseases in apricot trees, considering the computational resource constraints often encountered in practical applications, particularly the need for real-time detection on edge devices in the agricultural field, model lightweighting is especially important. By employing techniques such as network pruning and knowledge distillation, not only can the storage and computational requirements of the model be effectively reduced, but the performance of the model can also be maintained or even enhanced. This section provides a detailed description of where lightweight methods are utilized and how these methods are compatible with the apricot tree disease detection method proposed in this study.

Network pruning technology reduces the size and complexity of the model by removing non-critical connections (connections with small weights) or redundant neurons in the neural network. In this study, particularly within deep convolutional networks, structured pruning methods are adopted, which not only prune weights but also consider the importance of entire network layers. Specifically, by analyzing the contribution of each layer to the final detection task, convolutional layers or channels with lower contributions are selectively pruned. The mathematical expression of this method can be described by the following equation:

(20) $$L_{\mathrm{prune}}=L_{\mathrm{original}}+\lambda \sum_{l\in S}{∥W_{l}∥}_{p}$$

where $L_{\mathrm{original}}$ is the loss function of the original task, λ is the regularization parameter, S represents the set of layers selected for pruning, $W_{l}$ are the weights of layer *l*, and $∥W_{l}{∥}_{p}$ is the *p*-norm of the weights: commonly, p=1 for promoting sparsity through the L1 norm.

**Figure 6:**
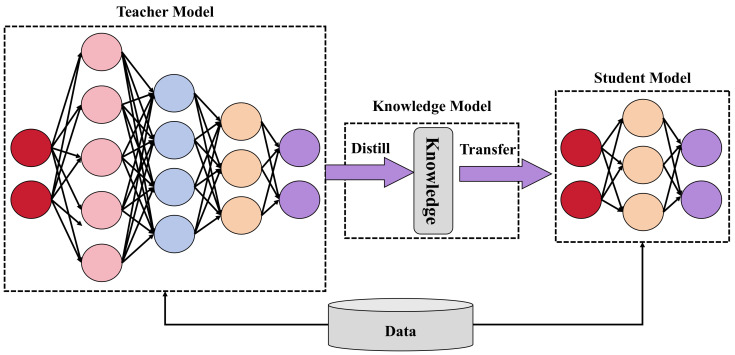
The illustration describes the basic principle of the knowledge distillation process. It shows the knowledge transfer mechanism from the “teacher model” to the “student model”. Typically, the teacher model is a large and complex pre-trained deep learning model that has already acquired extensive knowledge through training. During the knowledge distillation process, the output of the teacher model is used as guidance information (soft labels) to help train a smaller student model. The student model learns and extracts important knowledge by mimicking the output of the teacher model, achieving near-teacher-model performance while being smaller and more computationally efficient. This diagram clearly demonstrates the application framework of knowledge distillation technology, i.e., how to optimize the learning process of the student model through the teacher model.

Knowledge distillation is another lightweight strategy and is implemented by transferring the knowledge from a large and complex model (teacher model) to a smaller model (student model), as shown in Figure 6.

In this study, a smaller student network is designed, for which the structure is simplified, yet it is capable of learning key features from the teacher network. Knowledge distillation involves not only learning soft labels from the output layer but also includes feature distillation from intermediate layers, ensuring that the student model can capture feature representations similar to those of the teacher model. The loss function for distillation is:

(21) $$L_{\mathrm{distill}}=\left(1-\alpha \right)L_{\mathrm{original}}+\alpha T^{2}KL\left(\sigma \left(\frac{z_{t}}{T}\right),\sigma \left(\frac{z_{s}}{T}\right)\right)$$

where $z_{t}$ and $z_{s}$ are the output logits of the teacher and student models, respectively, σ denotes the softmax function, KL represents Kullback–Leibler divergence, *T* is the temperature parameter controlling the smoothness of the soft labels, and α is a coefficient balancing the original task loss and distillation loss.

Applying these lightweight techniques to the task of apricot tree disease detection significantly reduces the resource consumption when deploying models to edge devices, including computational resources and storage space. Moreover, by maintaining or even enhancing detection accuracy, the model becomes more efficient and accurate at real-time monitoring of diseases. Especially in the agricultural field, these advantages mean that advanced disease detection technologies can be deployed in a wider area—particularly, in remote and resource-limited regions—providing robust technical support for the prevention and control of apricot tree diseases.

### 3.5. Experimental Setup

#### 3.5.1. Hardware and Software Platform

Choosing an appropriate hardware and software platform is crucial when conducting deep learning research for apricot tree disease detection. Regarding hardware configurations, multiple servers have been equipped in the laboratory, each fitted with high-speed graphics processing units (GPUs): predominantly, NVIDIA’s Tesla V100 from New York and Quadro RTX 8000 series. These GPUs boast thousands of CUDA cores and substantial amounts of high-speed video memory and not only greatly enhance parallel processing capabilities but also significantly reduce the time required for model training and testing. The central processing units (CPUs) used are Intel Xeon Gold 6248R, which are high-performance CPUs designed for data centers and enterprise-level applications and feature 24 cores and 48 threads; they are capable of efficiently handling the demands of multitasking and concurrent execution. Each server is equipped with at least 128 GB of random access memory (RAM), ensuring smoothness and responsiveness when processing large-scale image data.

In terms of software configurations, a Linux operating system has been adopted to fully leverage the hardware capabilities, given its efficiency and stability when handling large datasets. Python 3.9.5 has been selected as the programming language due to its extensive support and rich library resources within the scientific computing and machine learning communities. Python’s flexibility and ease of use make the experimental code easy to write, test, and maintain. Regarding the choice of deep learning frameworks, this study primarily relies on PyTorch and TensorFlow 2.15.0, both of which provide advanced neural network building tools and automated gradient calculations, greatly facilitating the development and experimentation with complex models. PyTorch is particularly popular in academia due to its dynamic computation graph and intuitive programming paradigm, while TensorFlow is widely used due to its stability and scalability in production environments.

Moreover, OpenCV 4.1.0.25 and Pillow (PIL) 8.0 libraries are used for processing and analyzing image data. OpenCV offers a range of efficient image processing functionalities, such as cropping, scaling, and color conversion, which are crucial for preliminary image preprocessing. Pillow is used for reading and writing image files and supports multiple image formats. For data analysis and processing, NumPy 1.22.4 and SciPy 1.12.0, two scientific computing libraries, are employed. They provide robust mathematical operation capabilities such as matrix operations, statistical analyses, and optimization algorithms, which are essential for implementing complex data processing and model evaluation algorithms.

#### 3.5.2. Training Strategy

Setting appropriate hyperparameters is crucial for the learning efficiency and ultimate performance of deep learning models trained for apricot tree disease detection. First, the setting of the learning rate is discussed: this is a critical factor that controls the pace of model learning. In this study, the initial learning rate is set to 0.001 based on preliminary trials and recommendations in the literature. A smaller initial learning rate helps stabilize the descent at the beginning of training, avoiding the instability or premature convergence to local minima that may be caused by excessively large steps. As training progresses, a learning rate decay strategy is employed to finely adjust model weights and ensure quality convergence [53]. Specifically, if no significant decrease in loss on the validation set is observed for several consecutive epochs, the learning rate is automatically reduced by 10%. This adaptive adjustment method helps refine weight adjustments as the model approaches the optimal solution, thus enhancing model recognition accuracy.

Secondly, the choice of batch size is also an important consideration in model training. In this study, the batch size is set to 32 based on a balance between the available GPU memory and the complexity of the model structure. A larger batch size improves memory utilization and makes GPU parallel computing more efficient while also avoiding memory overflow due to excessively large batch sizes. Furthermore, a moderate batch size helps stabilize model learning, preventing inaccurate gradient estimates that can occur with too-small batches.

Regarding the choice of optimizer, given the efficiency of the Adam optimizer in handling non-convex optimization problems compared to traditional stochastic gradient descent (SGD) methods, Adam is selected as the primary optimization tool [54]. The Adam optimizer combines the advantages of momentum and adaptive learning rates and not only accelerates the convergence speed in the early stages of training but also automatically adjusts the learning rate for each parameter during training, adapting to the characteristics of the dataset. This is particularly important for dealing with the complexity and variability of apricot tree disease images.

For model evaluation methods, five-fold cross-validation is employed to assess the generalization ability of the model. Specifically, the dataset is randomly divided into five parts, with one part sequentially used as the test set and the others as the training set. This method maximizes the use of limited data resources and provides a fair assessment of model performance on different subsets, ensuring the reliability and stability of the evaluation results. Through cross-validation, detailed insights into the model’s performance under various conditions are obtained, allowing for timely adjustments to training strategies and model parameters.

#### 3.5.3. Performance Metrics

In the performance evaluation of deep learning models, precision, recall, accuracy, and mAP are critical indicators. Precision measures the accuracy of model predictions by indicating the proportion of samples correctly identified as diseased among those predicted as diseased. High precision signifies that most of the samples predicted as diseased are indeed diseased, which is crucial for reducing false actions in agricultural production.

Recall measures the proportion of diseased samples that the model correctly identifies, indicating the model’s ability to capture all actual diseased samples. High recall ensures that all potential diseases are identified, preventing missed diagnoses.

Accuracy represents the proportion of correct predictions among all predictions: including both diseased and non-diseased samples. In the context of apricot tree disease detection, high accuracy demonstrates that the model performs well across various sample types.

The mAP (mean average precision) is a comprehensive performance metric that is commonly used to evaluate the overall performance of object detection models and their balance across different categories. The mAP is calculated by averaging the precision and recall across various confidence thresholds, providing a thorough assessment of the model’s ability to handle multi-category detection tasks.

These metrics collectively provide a comprehensive performance evaluation, aiding with monitoring and improving model performance and ensuring the efficiency and accuracy of the apricot tree disease detection system in practical deployments.

### 3.6. Baseline Models

Choosing suitable baseline models is key in the research of apricot tree disease detection, as these models not only provide a standard for performance comparisons but also guide further model improvements. The selected baseline models include YOLOv5 [55], YOLOv8 [56], RetinaNet [57], EfficientDet [58], and Detection Transformer (DETR) [59], all of which have shown excellent performance in the field of computer vision: particularly in object detection tasks.

The YOLO series is renowned for its fast detection speed and real-time performance. YOLOv5 and YOLOv8 are newer versions in this series and continue to optimize the model architecture and detection efficiency. YOLO models treat object detection as a single regression problem: directly predicting bounding boxes and class probabilities from image pixels. This design significantly reduces model inference time, making it highly suitable for real-time applications. The network architectures of YOLOv5 and YOLOv8 include multiple convolutional layers, skip connections, and upsampling layers, which help capture image details at different scales. The loss function of the YOLO series combines coordinate loss, confidence loss, and classification loss:

(22) $$L_{\mathrm{total}}=\lambda_{\mathrm{coord}}L_{\mathrm{coord}}+\lambda_{\mathrm{conf}}L_{\mathrm{conf}}+\lambda_{\mathrm{class}}L_{\mathrm{class}}$$

where $L_{\mathrm{coord}}$ calculates the difference in coordinates between predicted and actual boxes, $L_{\mathrm{conf}}$ assesses the error in the confidence of whether a box contains an object, and $L_{\mathrm{class}}$ addresses classification errors. The λ coefficients balance the impacts of these loss components. RetinaNet is a single-stage object detection model that uses a feature pyramid network (FPN) and a focal loss function to address class imbalances, especially between background and foreground categories. RetinaNet’s FPN effectively utilizes multi-scale image information to enhance the detection capabilities for small objects. The core of RetinaNet is its innovative focal loss function, which adjusts the cross-entropy loss to reduce the weights of easily classified samples, enhancing the model’s focus on difficult samples:

(23) $$L_{\mathrm{focal}}=-\alpha {\left(1-p_{t}\right)}^{\gamma }\log\left(p_{t}\right)$$

where $p_{t}$ is the model’s predicted probability for the actual class, and α and γ are tuning factors to balance the contributions of positive and negative samples in the loss. EfficientDet is an exceptionally efficient object detection model that optimizes the dimensions of network scaling and uses a compound scaling method to uniformly adjust network width, depth, and image resolution. EfficientDet employs a weighted bidirectional feature pyramid network (BiFPN), allowing for efficient flow of easy-to-learn features. The loss function of EfficientDet, similar to other object detection models, includes coordinate and classification losses and is characterized by significantly reduced computational demands and model sizes while maintaining high accuracy. DETR redefines the approach to object detection using the Transformer architecture, eliminating the complex components commonly used in traditional detection models such as NMS. DETR directly formulates object detection as a set-prediction problem. Its loss function combines the confidence loss with outputs from the Hungarian matching algorithm, ensuring the model can effectively learn a set of objects rather than a sorted list.

These models were chosen as baselines due to their exemplary performance in the field of object detection and potential in handling practical applications such as agricultural disease detection. Comparing with these advanced models allows for a more comprehensive evaluation of the performance and practicality of the models proposed in this article. This not only helps validate the effectiveness of the new models but also provides insights into the challenges and limitations they may face in real-world applications.

## 4. Results and Discussion

### 4.1. Disease Detection Experimental Results

The design of this experiment aimed to validate and compare the performance of different advanced object detection models in the task of apricot tree disease detection and to demonstrate the effectiveness and superiority of the method proposed in this article. By comparing commonly used object detection models such as RetinaNet, EfficientDet, YOLOv5, DETR, YOLOv8, and the model proposed in this paper, a comprehensive evaluation of each model’s effectiveness was conducted based on four key performance metrics: precision, recall, accuracy, and mAP. The experimental results are presented in Table 2.

The method proposed in this article surpassed all the aforementioned models in all performance metrics: reaching a precision of 0.92, a recall of 0.89, an accuracy of 0.90, and an mAP of 0.91. Innovations in model design, loss function optimization, and training strategy significantly enhanced the performance of apricot tree disease detection. These improvements not only enable the model to perform excellently in processing single disease images but, more importantly, enhance the model’s adaptability and stability when facing complex and variable environments in practical applications. These research results indicate a promising future for the application of deep learning technology in the field of agricultural disease detection, providing valuable experience and a theoretical basis for related future research.

From the experimental results, significant improvements in disease detection performance were achieved using the proposed method compared to the performance of the benchmark models. Specifically, RetinaNet achieved a precision of 0.83, a recall of 0.80, an accuracy of 0.81, and an mAP of 0.82. RetinaNet, utilizing a feature pyramid and focal loss function, performs well on datasets with class imbalances but may not reach optimal performance in tasks requiring high local detail recognition such as apricot tree disease detection, possibly due to limitations of its inherent structure and loss function. EfficientDet [61] displayed slightly superior performance to RetinaNet, with a precision of 0.84, a recall of 0.82, an accuracy of 0.83, and an mAP of 0.84. EfficientDet’s innovative use of BiFPN and compound scaling techniques optimizes the learning of multi-scale features, which is highly beneficial for handling various sizes of disease spots on apricot trees. However, despite its strong performance, there is still room for improvement when processing images with highly complex backgrounds. YOLOv5 and YOLOv8 [62], as newer versions of the YOLO series, demonstrated superior performance. YOLOv5 recorded a precision of 0.85, a recall of 0.84, an accuracy of 0.85, and an mAP of 0.86; YOLOv8 achieved even higher marks with a precision of 0.89, a recall of 0.87, an accuracy of 0.88, and an mAP of 0.89. These results benefit from the YOLO model’s characteristics of being fast and accurate and its continually optimized model architecture, which provide excellent real-time and accurate performance. Particularly, YOLOv8’s further optimizations of network depth and width significantly improved its ability to capture fine disease features. The DETR model [63], with its unique Transformer architecture, also performed excellently in this set of experiments, achieving a precision of 0.87, a recall of 0.85, an accuracy of 0.86, and an mAP of 0.87. DETR eliminates complex components such as the NMS that is commonly used in traditional detection models and directly transforms the object detection problem into a set-prediction task; it proved more effective at detecting images with complex backgrounds and multi-scale targets. However, its training time is lengthy, and it demands relatively high computational resources.

### 4.2. Detection Results Analysis

The experimental design of this study aims to analyze the performance of different models for apricot disease detection through confusion matrices. Confusion matrices can detail each model’s classification accuracy across various disease categories, helping us understand the error rates for different categories and the models’ abilities to distinguish between different diseases.

**Figure 7:**
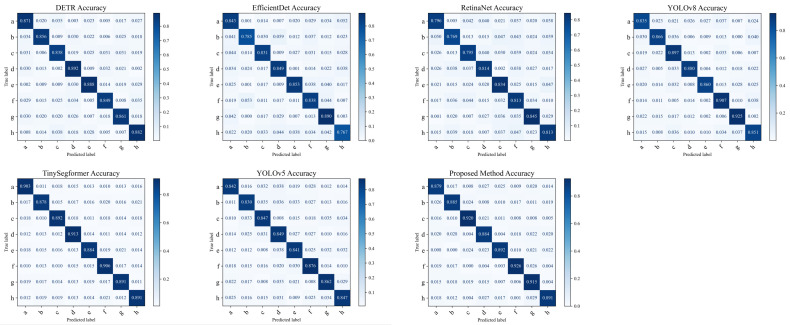
Confuse matrix of detection results: (a) Powdery Mildew Disease, (b) Brown Rot Disease, (c) Scab Disease, (d) Bacterial Leaf Spot Disease, (e) Almond Bee Disease, (f) Apricot Sore Disease, (g) Scale Chosomiasis Disease, and (h) Apricot Moth Disease.

As shown in Figure 7, the proposed method exhibits an average accuracy of 0.90 across all categories, demonstrating excellent performance. By combining the adaptive sampling latent variable network (ASLVN) and the spatial state attention mechanism, the model effectively handles multimodal data and balances between detailed image features and global information. Specifically, the ASLVN adjusts the model’s learning strategy according to different disease stages, while the spatial state attention mechanism further enhances the model’s focus on disease characteristics. From a mathematical perspective, the proposed method improves the detection accuracy of apricot diseases by integrating various advanced technologies. The ASLVN dynamically adjusts the sampling strategy, allowing the model to flexibly respond to different disease feature distributions and avoiding the limitations of traditional models in handling complex backgrounds and small targets. The spatial state attention mechanism assigns different weights to the feature map, enhancing the model’s attention to key areas and reducing misclassifications. Moreover, the proposed method effectively integrates image features and sensor data when handling multimodal data, ensuring high detection performance under different environmental conditions.

### 4.3. Application on Edge Computing Platform

The experimental design on edge computing platforms aims to validate the performance of the proposed apricot disease detection model across different hardware platforms, particularly focusing on its adaptability to and efficiency on resource-constrained environments. By testing on GPU platforms, Huawei P70 platforms, and Jetson Nano platforms, we can evaluate the model’s accuracy, model size, and frames per second (FPS) to determine its feasibility and performance in real-world applications. The experimental results show that on the GPU platform, the standard model achieves an accuracy of 0.90, a model size of 8.3 M, and 40.9 FPS. After applying knowledge distillation, the model’s accuracy slightly decreases to 0.86, the model size decreases to 3.7 M, and the FPS significantly increase to 68.4. This demonstrates that knowledge distillation can substantially reduce the model size and significantly improve processing speed while maintaining high accuracy. This trade-off is crucial in practical applications, especially in large-scale data scenarios requiring real-time processing, where miniaturization and efficiency can enhance system responsiveness and usability.

As detailed in Table 3, on the Huawei P70 platform, the standard model also achieves an accuracy of 0.90 with 13.6 FPS, while the knowledge-distilled model achieves an accuracy of 0.86 with 30.8 FPS. This again validates the advantage of knowledge distillation for improving model runtime efficiency. Despite a slight decrease in accuracy, the performance gain makes the model more suitable for environments with limited computational resources, such as mobile devices. On the Jetson Nano platform, the standard model achieves an accuracy of 0.90 with 9.1 FPS, while the knowledge-distilled model achieves an accuracy of 0.86 with 15.7 FPS. As a low-power edge computing device, the Jetson Nano has lower performance, but knowledge distillation significantly improves the model’s running speed, enabling effective operation in such a resource-constrained environment. From a mathematical perspective, knowledge distillation introduces the teacher model’s soft labels during training, allowing the student model to learn richer feature representations. This effectively reduces the number of model parameters while maintaining a high feature extraction capability. Additionally, the choice and testing of edge computing platforms demonstrates the performance differences of the model under various hardware conditions. On the GPU platform, due to its powerful computing capabilities, the model can run at a high frame rate. On mobile platforms and low-power edge devices, despite limited computing power, optimization techniques like knowledge distillation still achieve high operational efficiency and satisfactory accuracy. This indicates that the proposed model and optimization methods are highly flexible and adaptable in practical applications and can meet the needs of different application scenarios.

### 4.4. Results of the Adaptive Sampling Latent Variable Network Ablation Experiment

This experiment was designed to assess the impact and effectiveness of the ASLVN within the apricot tree disease detection model. By comparing two model configurations—one including the adaptive sampling latent variable network and the other without it—the specific contribution of adaptive sampling technology to model performance is vividly demonstrated. The experimental results, presented in Table 4, clearly reveal the importance of the ASLVN for enhancing the performance of apricot tree disease detection.

From the experimental results, it is observed that the model without the adaptive sampling latent variable network achieved a precision of 0.80, a recall of 0.77, an accuracy of 0.79, and an mAP of 0.79. In contrast, upon the introduction of the adaptive sampling latent variable network, all performance metrics showed significant improvement compared to the performance of the benchmark models: precision increased to 0.92, recall increased to 0.89, accuracy increased to 0.90, and mAP increased to 0.91. This substantial enhancement in performance can be fully explained and supported from both theoretical and practical perspectives. Theoretically, the ASLVN leverages the dynamic properties of the HMM, enabling dynamic adjustment of the sampling strategy and network parameters based on image features and historical information. This adaptability allows the model to more accurately capture the various stages and feature changes in disease development, thereby achieving higher recognition accuracy and response speed. For instance, when the model detects a potential early stage of disease in a region, the adaptive sampling mechanism can increase the sampling frequency of that area to ensure more local details are captured, enabling more accurate classification and diagnosis. Moreover, this dynamic adjustment mechanism can effectively handle variations in lighting, occlusion, and background noise, enhancing the model’s robustness in complex practical application scenarios.

From a practical perspective, the introduction of the adaptive sampling latent variable network significantly enhances the model’s ability to learn and generalize features of apricot tree diseases. In deep learning, sufficient data diversity and representativeness of training samples are crucial for improving model performance. By dynamically adjusting the sampling strategy, the adaptive sampling latent variable network not only optimizes the quality of data input but also reduces the risk of overfitting through the more efficient use of data. Additionally, the adaptive sampling mechanism can automatically identify and focus on those data features and samples that are most critical for enhancing the final performance, thereby making the training process more efficient and targeted. In summary, the ASLVN, through its highly flexible and dynamic characteristics, significantly improves the accuracy, efficiency, and robustness of the apricot tree disease detection model. This subnetwork not only has a solid theoretical foundation but also demonstrates clear performance advantages in practical applications, proving its practical value and broad application prospects for enhancing the performance of agricultural disease intelligent monitoring systems.

### 4.5. Results of the Spatial State Attention Mechanism Ablation Experiment

This experiment was designed to assess the impact of different types of attention mechanisms on the performance of an apricot tree disease detection model. By comparing models with no attention mechanism, a channel attention mechanism, a spatial attention mechanism, and a spatial state attention mechanism, the contributions of various attention strategies to enhancing model recognition accuracy were clearly demonstrated. The results, which are evaluated across four key metrics—precision, recall, accuracy, and mAP—provide a comprehensive performance analysis for disease detection.

According to the experimental results presented in Table 5, clear differences in performance levels were observed among the different attention mechanisms. In the absence of any attention mechanism, the model achieved a precision of 0.81, a recall of 0.79, an accuracy of 0.80, and an mAP of 0.80. This indicates that the model already possesses a baseline recognition capability without any attention assistance, yet there is room for improvement. Upon the introduction of the channel attention mechanism, all performance metrics improved: precision increased to 0.84, recall increased to 0.82, accuracy increased to 0.83, and mAP increased to 0.84. The channel attention mechanism, by emphasizing important feature channels, enhanced the model’s ability to capture key information, thereby improving performance. When the spatial attention mechanism was applied, the model’s performance further enhanced, with precision reaching 0.87, recall reaching 0.85, accuracy reaching 0.86, and mAP reaching 0.87. Spatial attention, by focusing on key areas within the image, optimized the model’s capacity to handle spatial information, which is particularly crucial for identifying diseases with localized features. Finally, upon the introduction of the spatial state attention mechanism, the model’s performance reached its peak, with precision increasing to 0.92, recall increasing to 0.89, accuracy increasing to 0.90, and mAP increasing to 0.91. This mechanism not only focuses on the spatial features of the image but also considers the state changes of the features, allowing the model to dynamically adjust its focus of attention and more effectively handle changes throughout the disease process, significantly improving the accuracy and stability of detection. From a theoretical and mathematical perspective, the advantage of the spatial state attention mechanism lies in its dual consideration of spatial information and temporal states. This mechanism, by establishing a state-dependent graph model, not only identifies key areas within the image but also adjusts its focus based on the stages of disease progression. For instance, it may focus more on the edges of leaves during the initial stages of disease and shift focus to changes in the center of the leaves as the disease progresses. Typically, the mathematical model utilizes a GCN combined with a state transition model and learns different state-dependent weight allocation strategies to achieve this dynamic and context-sensitive processing approach. This method allows the model to not only adapt to static images but also to effectively handle dynamic changes during disease progression, thereby exhibiting higher flexibility and accuracy in practical applications. In summary, through this series of ablation experiments, the specific contributions and roles of various attention mechanisms in the task of apricot tree disease detection are clearly demonstrated. The spatial state attention mechanism, with its unique advantage of considering both spatial and state information, greatly enhances the model’s recognition performance; particularly, it shows significant performance improvements in handling complex and dynamically changing disease images.

### 4.6. Future Work

Despite the significant achievements of this research in apricot tree disease detection, limitations exist, and future research directions require detailed discussion. First, although the model has demonstrated superior performance across several metrics, adaptability issues for specific scenarios persist. For instance, the model’s recognition accuracy may be impacted by extreme lighting conditions or when leaves are heavily obscured. This is primarily due to the limited presence of such extreme cases in the current datasets, which hinders the model’s ability to learn effective recognition capabilities under these conditions. Moreover, although the introduction of the ASLVN and the spatial state attention mechanism enhance the model’s ability to capture dynamic changes in diseases, the computational complexity of these advanced features is increased. This not only demands higher computational resources but may also limit the model’s deployment on resource-constrained devices. While model lightweighting techniques have been employed to address this issue, the lightweighting process may lead to performance degradation, particularly when the model needs to capture minute or complex disease features. Regarding the lack of diversity and representativeness in the datasets, future work could involve expanding the data collection scope to include more diverse samples: especially images of diseases under different lighting and occlusion conditions. This would not only enhance the model’s generalizability but also improve its adaptability to complex environments. Additionally, synthetic data augmentation techniques, such as generating a wider variety of disease images with GANs, could enrich the training dataset and improve the model’s robustness. In terms of model design, future research could explore more efficient network structures or optimization algorithms to reduce computational resource consumption while maintaining or enhancing performance. For example, more advanced network pruning techniques and parameter quantization methods could be investigated to further reduce the model’s resource requirements in practical applications. Additionally, exploring new lightweight network architectures, such as those based on depthwise separable convolutions, which have proven to be efficient and accurate in various vision tasks, could be beneficial.

Moreover, while the attention mechanisms implemented in this study have significantly improved the accuracy of disease detection, optimizing these mechanisms to adapt to a broader range of agricultural disease detection scenarios remains a subject for further research. Considering the latest developments in deep learning, such as Transformer network structures, could offer new solutions for handling more complex spatial and temporal data. Furthermore, future efforts could also focus on reducing the model’s reliance on labeled data while maintaining performance by exploring semi-supervised or unsupervised learning strategies, which are particularly important for agricultural applications, where data labeling is often costly. Finally, with the widespread adoption of IoT technology in agriculture, integrating the model into real-time monitoring systems to enable early warning and intelligent decision support for apricot tree diseases will be a crucial research direction. This requires that the model not only perform excellently on static images but also handle real-time data from various sensors and effectively integrate with other agricultural management systems. Through such integration, the level of intelligence in managing apricot tree diseases could be substantially enhanced, providing more scientific and precise support for agricultural production.

## 5. Conclusions

A comprehensive deep learning framework has been proposed in this paper with the aim of effectively addressing the challenge of accurately identifying and classifying diseases in apricot trees within complex natural environments. Disease detection is crucial for agricultural production, especially for ensuring crop health, preventing the spread of disease, and reducing economic losses. Advanced deep learning technologies, particularly the adaptive sampling latent variable network and the spatial state attention mechanism, significantly enhanced the model’s capability to recognize features of apricot tree diseases. Additionally, the implementation of model lightweighting techniques ensured feasibility and efficiency in practical applications. The main contributions and innovations of this study include: Firstly, we developed a deep-learning-based framework capable of effectively processing complex image data and accurately detecting apricot tree diseases. Secondly, we successfully integrated the adaptive sampling latent variable network, which is an innovative network structure that allows the model to dynamically adjust the learning process to accommodate different stages of disease progression. Furthermore, the introduction of the spatial state attention mechanism finely manages spatial information during the image processing process to effectively enhance the precision and recall of disease detection. Lastly, the implementation of a model lightweighting strategy ensures that the system is operational not only on high-performance computing platforms but also on resource-constrained mobile devices.

Experimentally, the method proposed in this paper performed excellently across several key performance metrics. Specifically, significant improvements were demonstrated in precision, recall, accuracy, and mAP compared to existing technologies. For instance, under configurations using the adaptive sampling latent variable network, the model achieved a precision of 0.92, a recall of 0.89, an accuracy of 0.90, and an mAP of 0.91. These results substantially exceed those configurations not utilizing this technology, fully proving the effectiveness of adaptive sampling in enhancing disease detection performance. Additionally, when comparing the effects of different attention mechanisms, the spatial state attention mechanism outperformed other mechanisms, including channel attention and basic spatial attention mechanisms, across all evaluation metrics. These outcomes not only validate the superiority of the spatial state attention mechanism in handling complex image data but also demonstrate its key role in enhancing overall model performance. These experimental results support the effectiveness of the methods used in this study and provide valuable experience and references for future research in this field. Overall, the research findings of this paper are not only innovative academically but also hold significant practical application value. Future work will explore new deep learning models and algorithms to further enhance the accuracy and real-time performance of apricot tree disease detection while extending these research findings to other crop disease detection fields, contributing to the development of smart agriculture.

## Figures and Tables

**Table 1:** Details of dataset used in this paper.

Disease Type	Number (before/after Dataset Augmentation)	Collection Location	Time
Brown Rot Disease	1398/1678	Xinhua Community in Linhe District, Bayannur, Su Dulun Town in Urad Front Banner, Yongqing County, Hebei Province	April–July 2023
Powdery Mildew Disease	926/1112	Shuguang Township in Linhe District, Bayannur, Su Dulun Town in Urad Front Banner	May–October 2023
Scab Disease	601/722	Xinhua Community in Linhe District, Bayannur, Pick-Your-Own Gardens in Changping District, Beijing	April–September 2023
Bacterial Leaf Spot Disease	882/1059	Yongqing County, Hebei Province, Shuguang Township in Linhe District, Bayannur	March–August 2023
Almond Bee Disease	1130/1356	Yongqing County, Hebei Province, Pick-Your-Own Gardens in Changping District, Beijing	March–October 2023
Apricot Moth Disease	1108/1330	Xinhua Community in Linhe District, Bayannur, Su Dulun Town in Urad Front Banner, Yongqing County, Hebei Province	May–July 2023
Scale Chosomiasis Disease	753/904	Shuguang Township in Linhe District, Bayannur, Yongqing County, Hebei Province	April–August 2023
Apricot Moth Disease	1209/1451	Yongqing County, Hebei Province, Su Dulun Town in Urad Front Banner	May–October 2023

**Table 2:** Disease detection results.

Model	Precision	Recall	Accuracy	mAP	Size	FPS
RetinaNet	0.83	0.80	0.81	0.82	33 M	21.6
EfficientDet	0.84	0.82	0.83	0.84	3.9 M	30.7
YOLOv5	0.85	0.84	0.85	0.86	7.5 M	42.5
DETR	0.87	0.85	0.86	0.87	41 M	18.3
YOLOv8	0.89	0.87	0.88	0.89	10 M	33.2
TinySegformer [60]	0.90	0.89	0.90	0.89	27 M	22.8
Proposed Method	0.92	0.89	0.90	0.91	8.3 M	40.9

**Table 3:** Results on different edge computing platforms.

Platform	Accuracy	Model—Size	FPS
GPU Platform	0.90	Normal—8.3 M	40.9
GPU Platform	0.86	Knowledge Distilled—3.7 M	68.4
Huawei P70	0.90	Normal—8.3 M	13.6
Huawei P70	0.86	Knowledge Distilled—3.7 M	30.8
Jetson Nano	0.90	Normal—8.3 M	9.1
Jetson Nano	0.86	Knowledge Distilled—3.7 M	15.7

**Table 4:** Subnetwork ablation experiment.

Model	Precision	Recall	Accuracy	mAP
Model with ASLVN	0.92	0.89	0.90	0.91
Model without ASLVN	0.80	0.77	0.79	0.79

**Table 5:** Different attention mechanisms ablation experiment.

Model	Precision	Recall	Accuracy	mAP
No attention mechanism	0.81	0.79	0.80	0.80
Channel attention mechanism	0.84	0.82	0.83	0.84
Spatial attention mechanism	0.87	0.85	0.86	0.87
Spatial state attention mechanism	0.92	0.89	0.90	0.91

## Data Availability

The data presented in this study are available on request from the corresponding author.
